# A Single Amino Acid in the Stalk Region of the H1N1pdm Influenza Virus HA Protein Affects Viral Fusion, Stability and Infectivity

**DOI:** 10.1371/journal.ppat.1003831

**Published:** 2014-01-02

**Authors:** Christopher R. Cotter, Hong Jin, Zhongying Chen

**Affiliations:** MedImmune LLC, Mountain View, California, United States of America; Johns Hopkins University - Bloomberg School of Public Health, United States of America

## Abstract

The 2009 H1N1 pandemic (H1N1pdm) viruses have evolved to contain an E47K substitution in the HA2 subunit of the stalk region of the hemagglutinin (HA) protein. The biological significance of this single amino acid change was investigated by comparing A/California/7/2009 (HA2-E47) with a later strain, A/Brisbane/10/2010 (HA2-K47). The E47K change was found to reduce the threshold pH for membrane fusion from 5.4 to 5.0. An inter-monomer salt bridge between K47 in HA2 and E21 in HA1, a neighboring highly conserved residue, which stabilized the trimer structure, was found to be responsible for the reduced threshold pH for fusion. The higher structural and acid stability of the HA trimer caused by the E47K change also conferred higher viral thermal stability and infectivity in ferrets, suggesting a fitness advantage for the E47K evolutionary change in humans. Our study indicated that the pH of HA fusion activation is an important factor for influenza virus replication and host adaptation. The identification of this genetic signature in the HA stalk region that influences vaccine virus thermal stability also has significant implications for influenza vaccine production.

## Author Summary

Influenza viruses cause seasonal epidemics and occasional pandemics, representing a threat to public health. The trimeric hemagglutinin (HA) surface glycoprotein mediates viral entry and plays important roles in viral host restriction, transmission and pathogenesis. The HA protein binds to the receptor on the cell via the head region, and mediates the low pH-triggered viral and cellular membrane fusion via the stalk region. The 2009 H1N1 pandemic (H1N1pm) viruses have been circulating in humans since 2009. A single amino acid change was found in the stalk region of recent H1N1pdm strains. In this study, we revealed that this amino acid change, by stabilizing the trimer structure, lowered the pH for fusion and exhibited a higher acid stability. Accordingly, the H1N1pdm with this change showed higher thermal stability after high temperature treatment. Furthermore, the H1N1pdm with this change had higher infectivity in ferrets, suggesting that recent H1N1pdm viruses have evolved to be more adapted in humans. Our data not only indicate the importance of the fusion activation pH to viral replication and host adaptation, but also provide solutions to improve the shelf-life of live vaccines in vaccine manufacture.

## Introduction

The swine-origin H1N1 2009 influenza virus (H1N1pdm) caused an estimated 151,700 to 575,400 deaths worldwide during the first 12 months of the 2009 pandemic [1], [2], [3]. Children and young adults were most vulnerable to infection because they lacked pre-existing immunity to the H1N1pdm virus [4]. This virus continues to be the predominant circulating H1N1 virus in the human population and has been a component of the annual seasonal influenza vaccine since the 2010 season.

Vaccination remains the most effective approach to prevent influenza virus infection. In response to the 2009 pandemic, a monovalent live attenuated influenza vaccine (LAIV) was produced and administered to millions of people. The LAIV is a 6∶2 reassortant virus that contains the six internal protein gene segments from the cold-adapted A/Ann Arbor/6/60 that confer the attenuation phenotype and the hemagglutinin (HA) and neuraminidase (NA) antigenic glycoprotein gene segments from A/California/7/2009 (Cal/09) H1N1pdm virus. However, the development of the H1N1pdm LAIV presented significant challenges. First, Cal/09-like viruses grew poorly in embryonated chicken eggs, the substrate used for vaccine manufacturing. The vaccine yield was improved by the E119K and G186D (H1 numbering) changes in the HA1 head region of the HA [5]. Second, the HA protein of Cal/09 could not be cleaved by bromelain for HA protein preparation. The insensitivity to bromelain cleavage is due to the HA 373 and 374 residues (HA2 position 46 and 47) in the HA stalk region [6]. Third, the Cal/09 LAIV has a shorter shelf-life at 4°C compared to the previous seasonal H1N1 vaccines, and the reason for this has yet to be determined.

The HA protein is a trimeric, class I membrane protein with a membrane-proximal stalk region and a membrane-distal receptor binding head region. It initiates viral entry by binding to sialic acid-containing receptors on the host cell surface. The receptor binding specificity of the HA protein has been known to be the major determinant of viral host tropism, pathogenicity and human to human transmission [7]. The cleavage of the HA precursor HA0 to the disulfide bond-linked HA1 and HA2 subunits by proteases in the host is required for viral fusion and infectivity. After viral internalization by endocytosis, the low pH in the endosome triggers an irreversible structural change in the HA protein, allowing the viral envelope to fuse with the endosomal membrane to release the ribonucleoprotein core of the virion into the cytosol [8]. The threshold pH for fusion activation and acid stability differs among different subtypes and strains and has been recognized as an important factor in viral host restriction, stability, transmissibility and pathogenesis [3], [9], [10], [11], [12], [13].

Recent surveillance data has revealed the emergence of a prominent mutation, E47K (HA2 numbering) in the HA2 stalk region of H1N1pdm isolates [14], [15], [16], [17]. In this study, we compared two H1N1pdm viruses that differ at the HA2 position 47 of their HA proteins. We demonstrated that the HA2-47 residue determined the threshold pH of fusion. The inter-monomer interaction between HA2-47 and the residue at HA1 position 21 in the stalk region of the HA protein was identified as the structural basis for the different pH for fusion activation and thermal stability of H1N1pdm viruses. Furthermore, H1N1pdm viruses with HA2-K47 are more infectious in ferrets, underscoring the importance of the fusion activation pH of the HA protein for viral host adaptation and fitness.

## Results

### The HA2-47 residue in the H1N1pdm HA stalk region affects the threshold pH for membrane fusion

**Figure 1:**
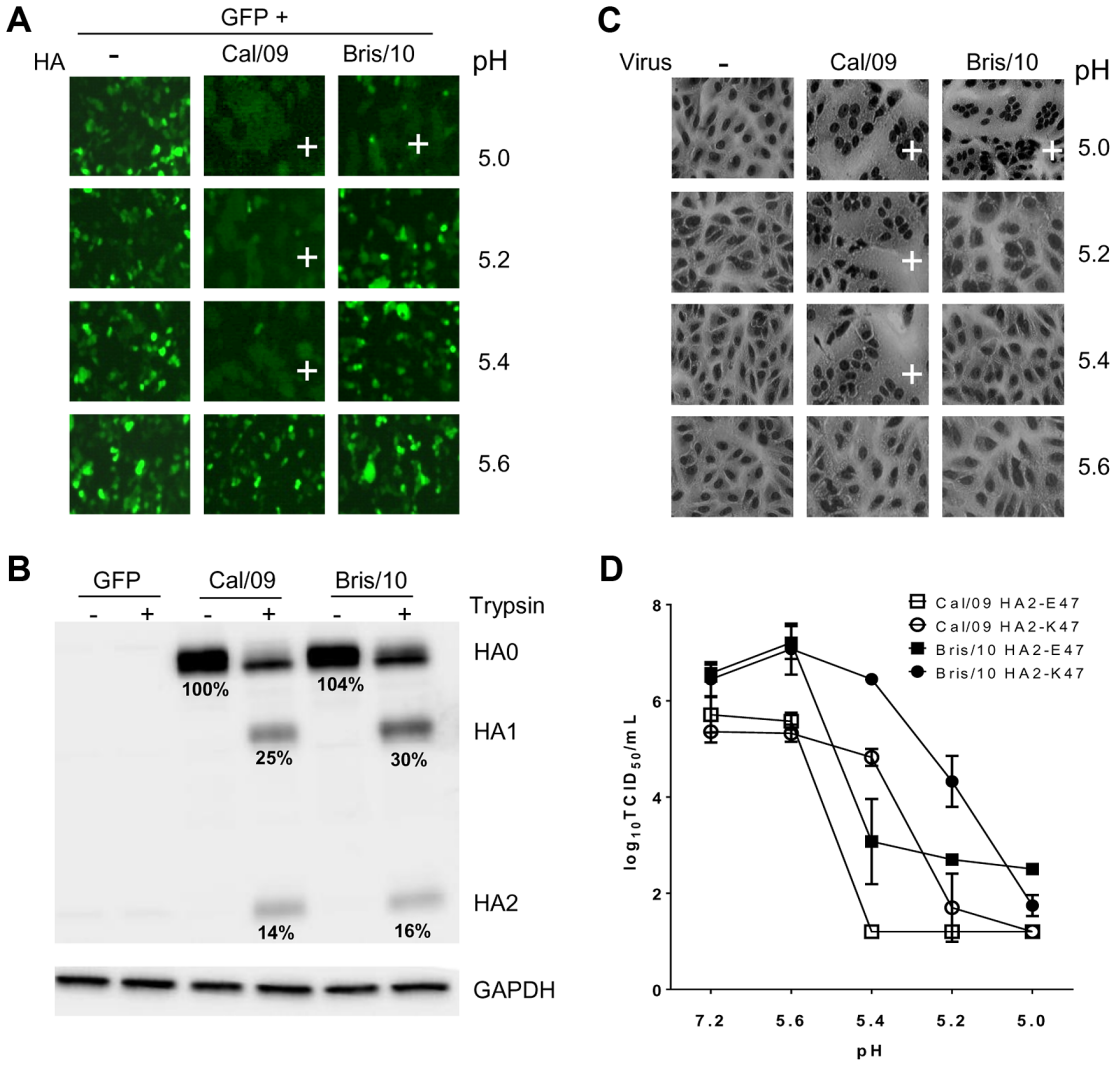
Cal/09 and Bris/10 HA differ in the threshold pH for membrane fusion. (**A**) Membrane fusion in 293T cells expressing Cal/09 or Bris/10 HA proteins. 293T cells were transfected with a GFP expression plasmid (−), a dual expression plasmid co-expressing GFP and Cal/09 HA or GFP and Bris/10 HA. Membrane fusion was observed after incubation with the indicated pH buffers. “+” indicates the fused cells. (**B**) Expression of the HA proteins in the transfected 293T cells. Both cleaved (HA1 and HA2) and uncleaved (HA0) forms of the HA proteins were detected by western blot using a sheep anti-HA polyclonal antibody. Values below the HA0 bands (−) trypsin represent the relative amount of Cal/09 and Bris/10 HA proteins. Values below the HA1 and HA2 bands (+) trypsin indicate the percentage of HA1 and HA2 to total HA (HA0+HA1+HA2). All the values are the average of three independent experiments. (**C**) Membrane fusion in Vero cells infected with Cal/09 or Bris/10 viruses. Vero cells were either mock-infected (−) or infected with Cal/09 or Bris/10 viruses at an MOI of 4.0. At 14 hours post infection, cell membrane fusion was triggered as above. “+” indicates syncytia formation. (**D**) Viral infectivity titers of wt Cal/09 and Bris/10 viruses (HA2-E47 or HA2-K47) after treatment with the indicated pH buffers.

The HA proteins of the original H1N1pdm strain A/California/7/2009 (Cal/09) and a later strain A/Brisbane/10/2010 (Bris/10) were evaluated for their membrane fusion activity by a transient expression assay. 293T cells were transfected with a dual-promoter expression plasmid expressing both GFP and HA. After 24 hours of transfection, the cells were treated with trypsin to allow HA cleavage, followed by incubation in various pH buffers ranging from pH 5.6 to 5.0 to trigger membrane fusion. In contrast to the cells expressing only GFP, the cells expressing both GFP and HA proteins underwent membrane fusion at pH 5.0 (Figure 1A), which was apparent by the diffusion of the GFP signal. Cells transfected with the HA from Cal/09 and Bris/10 exhibited a 0.4 pH unit difference in the threshold pH at which fusion occurred. The fusion of the Cal/09 HA was triggered at pH 5.4, while that of Bris/10 required pH 5.0. The levels of HA expression (100% vs. 104%) and the ratio of the cleaved HA1/HA2 proteins (39% vs. 46%) after trypsin treatment were comparable between the Cal/09 and Bris/10 HA-transfected cells (Figure 1B).

The difference in the pH required for fusion between the HA proteins of the two H1N1pdm viruses was further confirmed using a viral fusion assay (Figure 1C). Vero cells were infected with wild type (wt) Cal/09 or Bris/10 followed by a low pH treatment to trigger membrane fusion as measured by syncytia formation. Consistent with the transient expression assay, the Cal/09 virus exhibited a higher threshold pH for fusion (pH 5.4) than the Bris/10 virus (pH 5.0).

**Figure 2:**
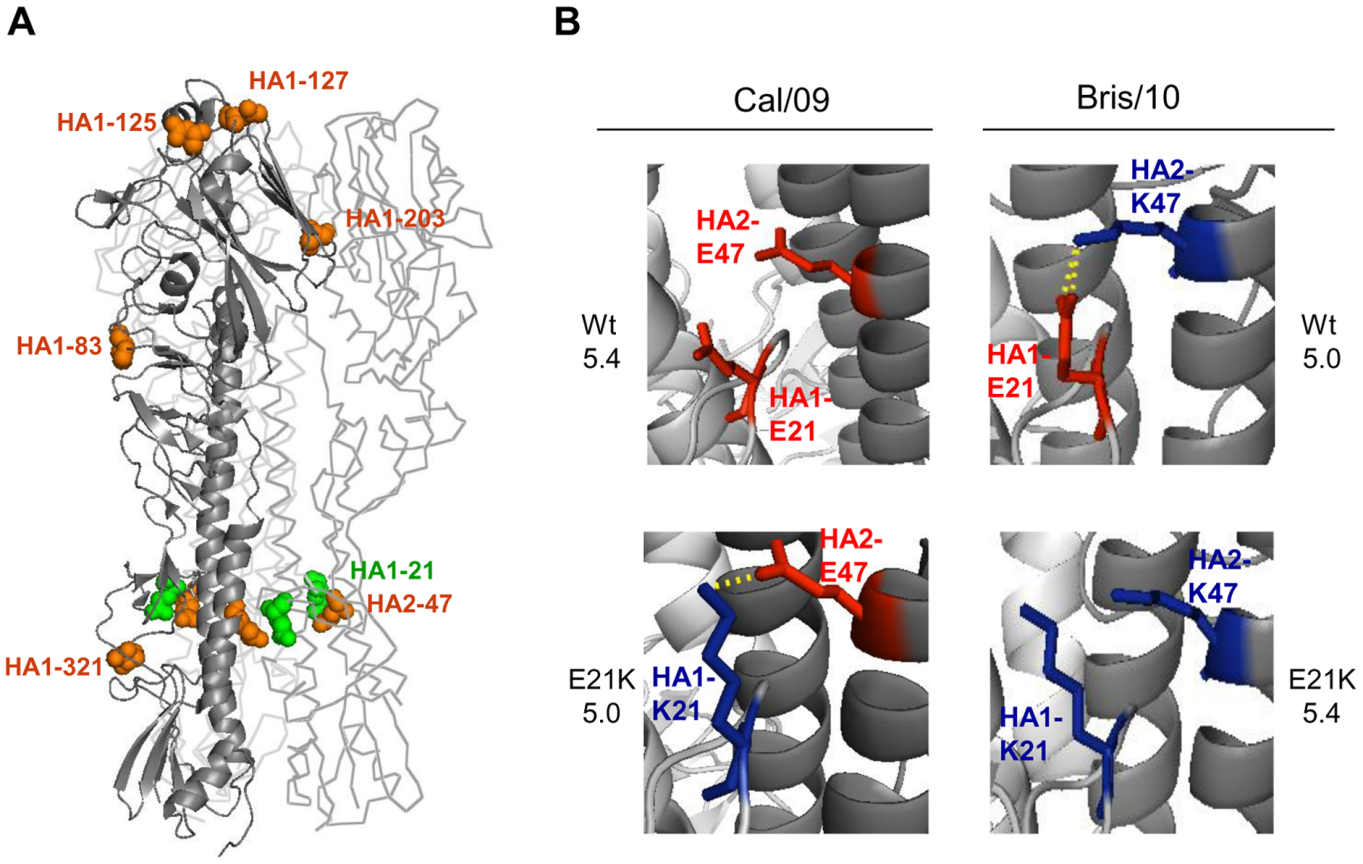
An inter-monomer interaction between the HA residues HA1-21 and HA2-47 determines the threshold pH for fusion. (**A**) The Cal/09 HA trimer structure (PDB file: 3LZG) was analyzed using Py-Mol modeling software. The six amino acid differences between the Cal/09 and Bris/10 HA proteins are highlighted in orange. The conserved amino acid HA1-21 is highlighted in green. The locations of the three HA1-21 and HA2-47 residues in each monomer are indicated. Only the location in one monomer is shown for the other residues. (**B**) Structure modeling of residues HA1-21 and HA2-47, salt bridges between HA1-E21 and HA2-K47 (Bris/10), or HA1-K21 and HA2-E47 (Cal/09 E21K) are indicated. The threshold fusion pH of each combination, as determined by the GFP fusion assay, is indicated adjacent to each model.

There are 6 amino acid differences between the HA proteins of Cal/09 and Bris/10 in the HA head and stalk region (Figure 2A). Each of the Bris/10-specific residues were introduced into the corresponding position of the Cal/09 HA gene, and the threshold pH for fusion was determined by the HA/GFP co-expression fusion assay (Table 1). Only the substitution of the glutamic acid at HA2 position 47 to lysine (Cal/09 HA2-E47K) effectively lowered the threshold pH of the Cal/09 HA from 5.4 to 5.0. Conversely, the HA2-K47E substitution in the HA of Bris/10 raised the threshold pH for fusion from 5.0 to 5.4. These results indicate that HA2 residue 47 regulates the threshold pH for fusion in the H1N1pdm strains.

**Table 1:** Mapping the HA residues that affect the threshold pH for membrane fusion.

HA	HA amino acid position						Threshold pH for fusion
	HA1	HA1	HA1	HA1	HA1	HA2	
	83	125	127	203	321	47	
Bris/10	S	D	E	T	V	K	5.0
Cal/09	P	N	D	S	I	E	5.4
Cal/09 M1	S	-	-	-	-	-	5.4
Cal/09 M2	-	D	-	-	-	-	5.4
Cal/09 M3	-	-	E	-	-	-	5.4
Cal/09 M4	-	-	-	T	-	-	5.4
Cal/09 M5	-	-	-	-	V	-	5.4
Cal/09 M6	-	-	-	-	-	K	**5.0**
Bris/10 M1	-	-	-	-	-	E	**5.4**

The different amino acids between the HA of Cal/09 and Bris/10 are listed. Single amino acid substitution was introduced into the Cal/09 HA plasmid (-: no change). The threshold pH for fusion was determined by the HA/GFP co-expression fusion assay.

Recombinant wt Cal/09 and Bris/10 that differed at the HA2-47 residue were generated and tested for their susceptibility to acid inactivation (Figure 1D). Viral infectivity was kept at the starting titers (approximately 6.0 log_10_ TCID_50_/mL) in the pH range of 7.2 to 5.6. In contrast, at pH 5.4, the infectious viral titers of Cal/09 HA2-K47 mutant and wt Bris/10 with HA2-K47 were more than 3.0 logs higher than their counterparts containing HA2-E47. By pH 5.0, the infectivity of all viruses dropped to only 2.0 log_10_ TCID_50_/mL. The data demonstrated that the HA2-47 residue affected the threshold pH for fusion and the acid stability of the viruses.

### The interaction between HA2-47 and HA1-21 affects the threshold pH for fusion

HA2 residue 47 is in the subunit interface of the HA trimer, which undergoes conformational changes during membrane fusion. It has been predicted that the HA2-E47K change introduces an inter-monomer salt bridge between the glutamic acid residue at HA1 position 21 (E21) from one monomer to the HA2-K47 in another (Figure 2) [17], [18], [19]. The HA1-E21 residue is highly conserved among the H1 subtype viruses, including Cal/09 and Bris/10. We hypothesized that the inter-monomer interaction between HA1-E21 and HA2-K47 in the Bris/10 HA trimer, which is not found in the Cal/09 trimer due to the presence of HA2-E47, results in a more acid-stable structure than that found in the Cal/09 trimer, such that a lower pH is required for the Bris/10 HA to undergo the conformational change required for viral infection compared to the Cal/09 HA. To confirm that the HA1-E21 and HA2-K47 inter-monomer interaction was critical for the threshold pH of fusion, an HA1-E21K change was introduced into the HA of Cal/09 (Cal/09 HA1-E21K) and Bris/10 (Bris/10 HA1-E21K), and the threshold pH for fusion was determined by the HA/GFP co-expression fusion assay. As predicted, the HA1-E21K change in Cal/09 lowered the threshold pH for fusion from 5.4 to 5.0, which can be explained by the introduction of the stabilizing salt bridge between HA1-K21 and HA2-E47. Conversely, Bris/10 HA1-E21K, which eliminated the interaction between residues HA1-21 and HA2-47 in the Bris/10 HA, raised the threshold pH for fusion from 5.0 to 5.4 (Figure 2). These data provide direct evidence that the interaction between HA residues HA1-21 and HA2-47 stabilize the HA trimer structure, resulting in a lower pH for fusion.

### Viruses with a higher threshold pH for fusion grow better in Vero cells

**Figure 3:**
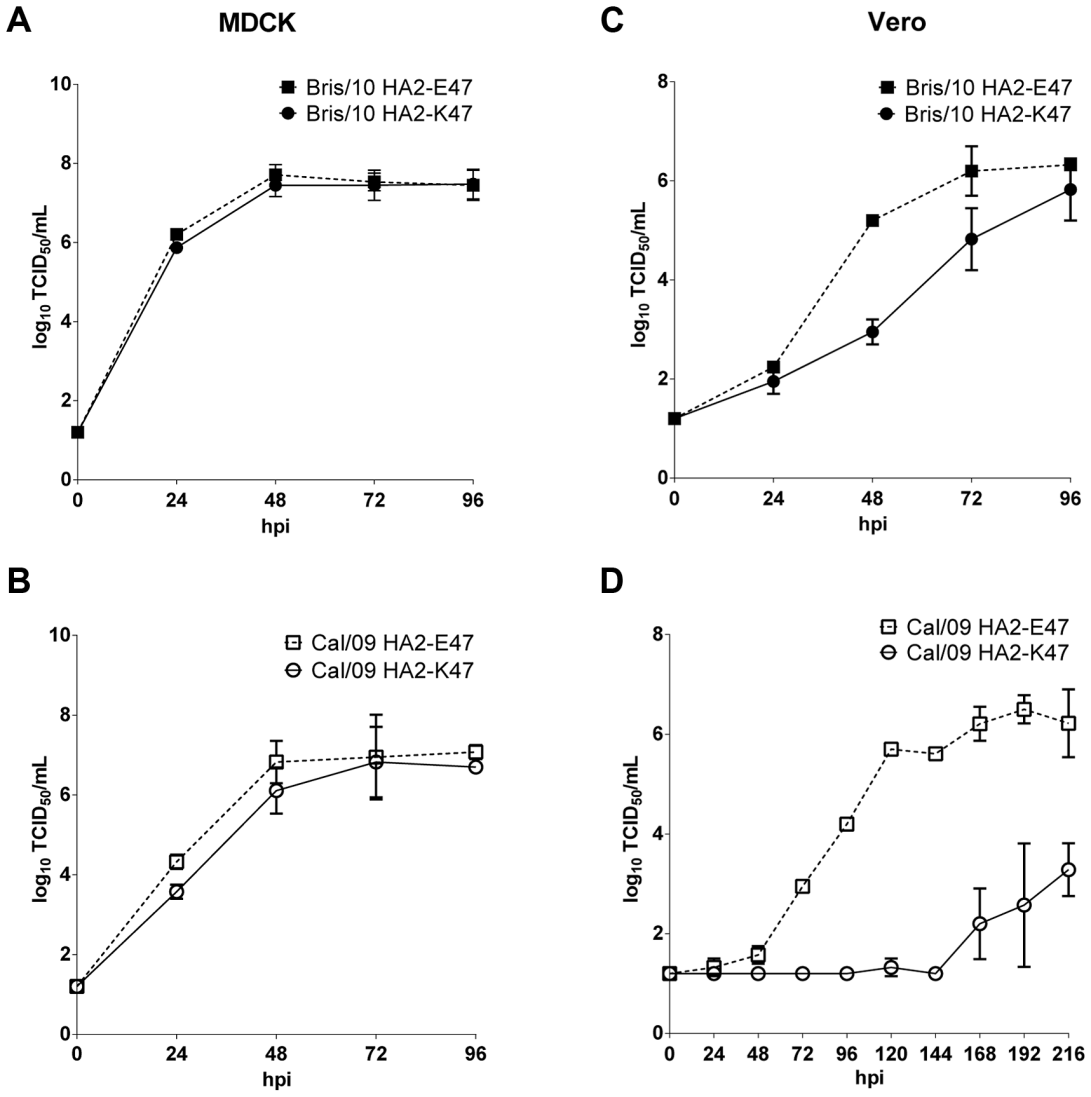
Growth kinetics of H1N1pdm viruses with HA2-E47 or K47 in MDCK and Vero cells. MDCK (A, B) and Vero (C, D) cells were infected with recombinant wt Bris/10 (HA2-K47) and the HA2-E47 mutant (A, C), or wt Cal/09 (HA2-E47) and the HA2-K47 mutant (B, D) at an MOI of 0.004. At the indicated time of post infection, virus titers were determined by TCID_50_ in MDCK cells.

Cell lines that differ in their endosomal pH may support the replication of viruses with different pH thresholds for fusion differently. Vero cells have been reported to have a higher endosomal pH than MDCK cells [20]. To test the effect of viral fusion pH on virus growth, the growth kinetics of wt Bris/10 and wt Cal/09 viruses that differed at the HA2-47 residue were compared in Vero and MDCK cells (Figure 3). All viruses tested grew efficiently with similar kinetics in MDCK cells and reached similar peak titers (Figure 3A and B). In contrast, the two viruses with the high threshold pH of fusion (wt Cal/09 HA2-E47 and the Bris/10 HA2-E47 mutant virus) achieved peak titer with faster kinetics in Vero cells than the corresponding viruses containing HA2-K47 (Figure 3C and D). Remarkably, the Cal/09 virus with the single HA2-E47K mutation exhibited a greater defect in replication. No viral titers were detected until as late as 186 hpi. Thus, a higher endosomal pH in Vero cells may not be able to support the replication of viruses with a fusion pH threshold of 5.0 as efficiently as the one with a fusion pH threshold of 5.4.

### The HA2-47 residue affects the thermal stability of H1N1pdm viruses

**Figure 4:**
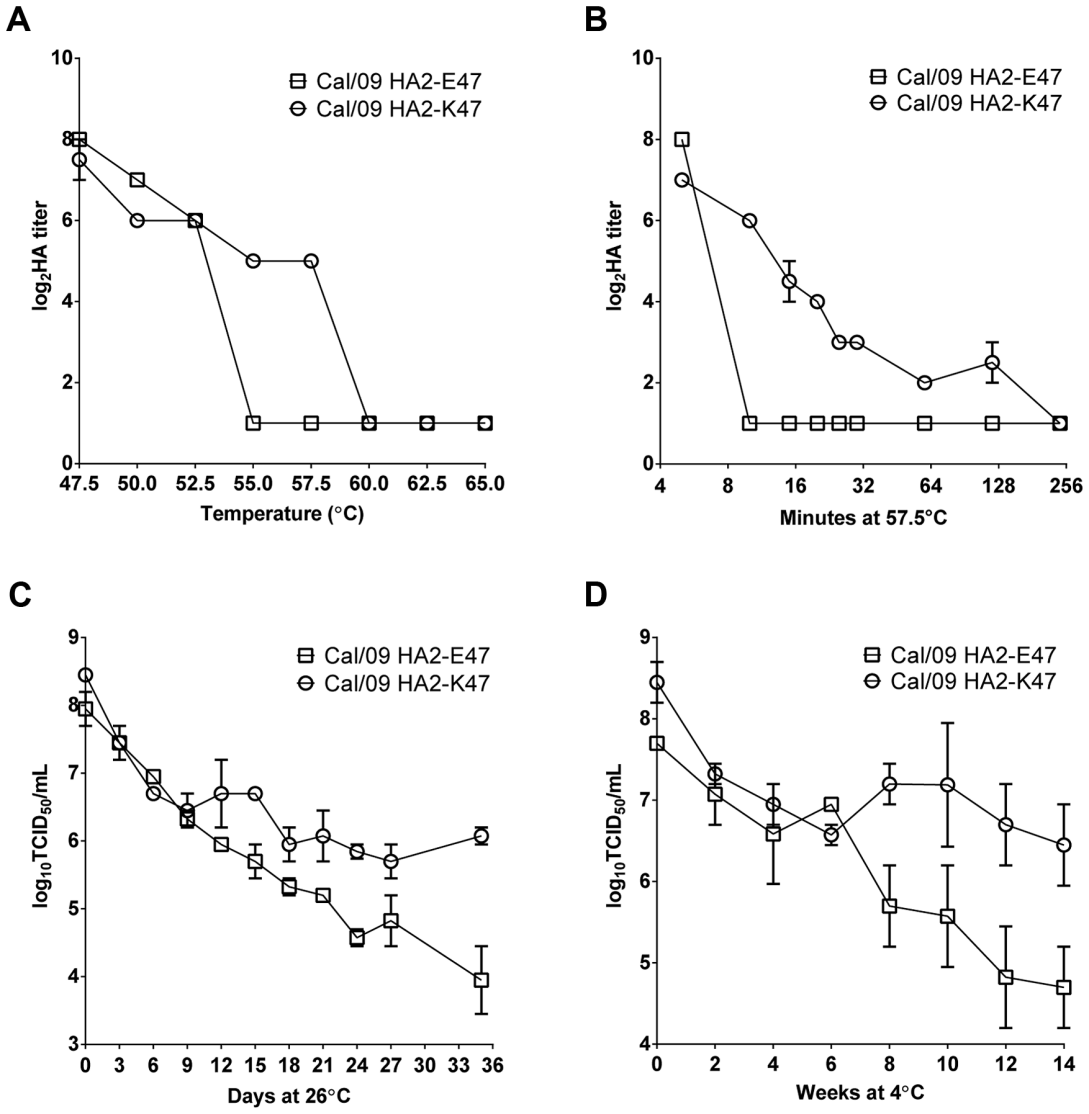
The impact of HA2-47 on the thermal stability of H1N1pdm vaccine viruses. The 6∶2 *ca* viruses with equalized HA titers were incubated at the indicated temperatures. Following either a 20 minute incubation at the indicated high temperatures (A) or a 4 hour time-course at 57.5°C (B), the HA titers were re-measured. The 6∶2 *ca* viruses were purified and re-suspended in 1× SP-cGAG. Duplicate aliquots of virus were maintained within a controlled chamber at either 26°C for 35 days (C) or 4°C for 14 weeks (D). At the indicated time points, the infectivity of viruses was examined by TCID_50_ assay in MDCK cells.

Both low pH and high temperature treatment can induce an irreversible conformational change in the HA protein, resulting in the formation of an inactivated fusogenic structure [21], [22], [23]. To determine if the high threshold pH for fusion (low acid stability) correlates with the low thermal stability of the Cal/09 vaccine virus, aliquots of Cal/09 HA2-E47 and Cal/09 HA2-K47 were incubated at high temperatures, and the integrity of the HA protein was assessed by a hemagglutination assay. Following a 20-minute incubation at 47.5–65°C, both viruses retained HA titer after incubation at temperatures of up to 52.5°C (Figure 4A). The Cal/09 HA2-E47 virus showed a precipitous drop in HA titer following the incubation at 55°C. In contrast, the Cal/09 HA2-K47 virus retained HA titer at 55°C, not showing a similar drop until 60°C, a temperature of 5°C higher than its HA2-E47 counterpart. Virus stability was then examined by holding the Cal/09 virus pair at 57.5°C over a time period of 240 min (Figure 4B). Cal/09 HA2-K47 had a more gradual decline in HA titer over time compared to Cal/09 HA2-E47, demonstrating that the Cal/09 HA2-E47 virus with lower acid stability also displayed lower thermal stability compared to the HA2-K47 counterpart.

To investigate whether the HA2-47 residue affects the shelf-life of the Cal/09 vaccine in vaccine formulation, Cal/09 HA2-E47 and Cal/09 HA2-K47 vaccine viruses were purified and prepared in the vaccine buffer formulation with 1× SP-cGAG. The viruses were incubated at 26°C (Figure 4C) and 4°C (Figure 4D), and the virus infectivity was measured at 3-day and 2-week intervals, respectively. The Cal/09 HA2-K47 vaccine virus maintained a higher titer over time than the Cal/09 HA2-E47 virus under both temperature conditions. The HA2-K47-containing virus was 130-fold more infectious when held at ambient temperature (Figure 4C) and 50-fold more infectious after more than 3 months at 4°C than the HA2-E47-containing virus (Figure 4D). Taken together, these data show that the HA2-E47K mutation in Cal/09 conferred greater temperature stability across temperatures from 4°C to 57.5°C.

### H1N1pdm viruses with HA2-K47 are more infectious in ferrets

The global emergence and increased prevalence of the HA2-E47K change in human H1N1pdm isolates have been reported [15], [16], [17], [24]. To evaluate whether increased stability due to HA2-E47K conferred a fitness advantage to the H1N1pdm virus, ferret studies were carried out to compare the infectivity of the two pairs of H1N1pdm viruses: wt Cal/09 HA2-E47 and the Cal/09 HA2-K47 mutant; and wt Bris/10 HA2-K47 and the Bris/10 HA2-E47 mutant. Ferrets were inoculated with three doses of 10, 100 or 1000 PFU of each virus. As shown in Table 2, with high virus inputs (100 and 1000 PFU), nearly every animal became infected. All of the viruses were detected in the respiratory tissues, and the ferrets exhibited weight loss and fever (data not shown). The GMT titers of the shed viruses were not significantly different between K47 and E47 viruses. In addition, the antibody titers induced by the viruses that differed at position HA2-47 were also comparable (data not shown). Interestingly, animals infected with the Bris/10 pair shed virus at higher titers than animals infected with the Cal/09 pair, indicating that other HA residues different between Bris/10 and Cal/09 affected viral shedding.

**Table 2:** The impact of the HA2-47 residue on wt H1N1pdm infectivity in ferrets.

Virus	Infection dose (PFU)	No. infected/total	Nasal wash virus titer (log_10_TCID_50_/mL±SD)	FID_50_ (PFU)
Cal/09 HA2-E47	10	1/4	6.58±0	17.8
	100	4/4	3.62±1.23	
	1000	4/4	2.95±0	
Cal/09 HA2-K47	10	4/4	3.87±0.76	3.2
	100	4/4	2.82±0.76	
	1000	4/4	2.12±0.14	
Bris/10 HA2-K47	10	6/7	6.11±0.7	4.4
	100	7/7	5.97±0.7	
	1000	7/7	5.35±0.48	
Bris/10 HA2-E47	10	2/7	6.83±0.19	23
	100	6/7	6.04±0.67	
	1000	7/7	5.59±0.4	

Groups of ferrets were inoculated with the indicated amount of wt A/California/7/09 (Cal/09 HA2-E47), the E47K mutant (Cal/09 HA2-K47), wt A/Brisbane/10/2010 (Bris/10 HA2-K47) or the K47E mutant (Bris/10 HA2-E47). The number of infected ferrets in each group (N = 4 or 7) and geometric mean titers of the nasal wash samples from the infected ferrets at day 3 post-infection determined by TCID_50_ are indicated. PFU: plaque forming units. FID_50_: 50% ferret infection dose; SD: standard deviation.

At the low dose of infection (10 PFU), both viruses with HA2-K47 were highly infectious in ferrets (Cal/09 HA2-K47 mutant, 4/4 infected and wt the Bris/10 HA2-K47, 6/7 infected). In contrast, the wt Cal/09 HA2-E47 virus infected only one of four ferrets at the 10 PFU dose. The Bris/10 HA2-E47 mutant virus did not infect any ferrets in study 1 (N = 3) and infected two out of four ferrets in study 2 (N = 4). The FID_50_ of the wt Cal/09 HA2-E47 virus was calculated to be 17.8 PFU, approximately 5 times higher than the mutant Cal/09 HA2-K47 (3.2 PFU FID_50_). Similarly, the FID_50_ of the wt Bris/10 HA2-K47 virus was calculated to be 4.4 PFU, 5 times lower than that of Bris/10 HA2-E47 (23 PFU FID_50_). The differences among the FID_50_ data was significant based on the 95% confidence intervals using the Spearman-Karber method. Thus, the HA2-E47K change in recently circulating H1N1pdm viruses increased viral infectivity in ferrets.

## Discussion

The original A/California/7/09 (Cal/09)-like H1N1pdm strains contained E47 in the HA2 stalk region. Since July 2009, an E47K mutation emerged and rapidly became predominant worldwide. Our studies have demonstrated the importance of this residue for viral membrane fusion activity, acid and thermal stability and infectivity *in vivo*. The pH for fusion activation has been shown to regulate the virulence and transmission of influenza H5N1 viruses in ferrets and mice [11], [13]. Our study on H1N1pdm supports the model that the pH of fusion activation is an important factor in determining viral fitness in humans.

The HA2-47 residue is located in the helix region near the fusion peptide which undergoes a dramatic conformational change to form a coiled-coil structure during the low pH triggered membrane fusion. It mediates an electrostatic interaction to other residues in the HA trimer interface to maintain a structure required for membrane fusion [8], [19], [25]. The HA2 Q47R and Q47K changes in the H3 and H7 viruses, respectively, have been previously reported to affect virus fusion pH [26]. Structure modeling has suggested that a salt bridge between a highly conserved acidic residue HA1-E21 and the basic residue HA2-K47, which is absent between HA1-E21 and HA2-E47, would require higher protonation (a lower pH) for fusion to occur. We experimentally confirmed that the interaction between the residues HA1-21 and HA2-47 in the HA monomer interface regulates the pH of fusion activation for H1N1pdm viruses. High temperature at neutral pH can also induce a conformational change in the HA that was indistinguishable from the low pH-induced conformational change, in which the metastable prefusion HA changes to a fusogenic form leading to protein inactivation [21], [22], [23]. Thus, a higher threshold pH for fusion normally reflects a lower viral thermal stability. We showed that the viruses containing HA2-K47 exhibited higher stability after heat treatment or prolonged incubation at 26°C and 4°C compared to their HA2-E47 counterparts. It is noteworthy that each subtype of influenza viruses contains specific and highly conserved amino acids at positions HA1-21 and HA2-47 of the HA. It would be interesting to further explore the role of positions HA1-21 and HA2-47 in viral fusion activity in other influenza virus subtypes.

The inter-monomer interaction between residue 21 in the HA1 region and residue 47 in the HA2 region identified in this study provides a structural explanation for the decreased thermal stability of the H1N1pdm vaccine viruses at 4°C, a challenge encountered during the 2009 H1N1 pandemic response. The H1N1pdm vaccine strain with the HA2-K47 residue is preferred as a vaccine candidate because of its prolonged shelf-life at 4°C. It has also been confirmed at MedImmune that the Bris/10 (HA2-K47) monovalent LAIV has an improved shelf-life at 4°C compared to Cal/09 (HA2-E47) (data not shown). The HA2 residue 47 was also responsible for the poor cleavage of the Cal/09 HA by bromelain, an enzyme used to release the HA ectodomain from the viral envelope. Bris/10 with HA2-K47 can be efficiently cleaved by bromelain, while Cal/09 with HA2-E47 cannot [6].

Virus adaptation to different cell lines, host species or under the pressure of higher endosomal pH induced by the antiviral drug amantadine often lead to mutations in the HA that result in a change in the fusion pH [26], [27], [28]. Consistent with a previous report that a PR8 HA2 mutant with a higher HA fusion pH threshold of 5.4 grew better in Vero cells than the wt PR8 with a lower fusion pH threshold of 5.2 [20], we observed a growth advantage forH1N1pdm viruses with HA2 E47, which have a fusion pH threshold of 5.4, in Vero cells. A more recent H1N1pdm isolate from 2012 containing K47 was also tested and found to have a similar growth defect in Vero cells (data not shown). The engineering of HA with an optimal pH for fusion activation resulting in higher viral replication could be leveraged to improve virus yield for cell culture-based vaccine production.

An optimal fusion pH is required for balancing viral acidic stability in the mildly acidic nasal tissue environment and fusion activation in the acidic endosome. Human influenza viruses have a lower fusion pH than their avian counterparts, indicating that the pH of fusion activation may influence viral transmission and establishment in new species [10]. Studies on H5N1 have highlighted the importance of the pH of fusion activation to host-specific replication and pathogenicity. Only a narrow pH range of 5.5–6.2 supports efficient and sustainable infection of H5N1 virus in ducks [29], [30]. The pathogenicity of highly pathogenic avian influenza virus (HPAI) H5N1 in chickens was associated with a higher fusion pH compared to moderately pathogenic avian influenza (MPAI) H5N1 [9]. A K58I mutation in the HA2 of H5N1 that decreased the activation pH for fusion reduced viral pathogenicity in ducks, but increased the virulence and the infectivity and immunogenicity of an intranasal H5N1 vaccine virus in mice, showing the species specific impact of the fusion activation pH [13], [29], [31], [32]. For avian H5N1 viruses to transmit among ferrets, sequence changes are required in both the HA head region to alter receptor binding specificity and the stalk region, in which a single residue change (T318I) near the fusion peptide lowers the pH for fusion and increases virus stability [11]. Therefore, in addition to the receptor binding preference conferred by the HA protein and viral replication capability attributed by the internal protein genes [33], the pH of HA fusion activation is another important factor for influenza virus host tropism and transmission.

A mouse-adapted H1N1 virus that acquires an HA2-W47G change was reported to lower both the fusion pH and lethal dose in mice [34], [35]. The E47K change at the same HA2 position in the H1N1pdm viruses resulted in a similar biological phenotype for fusion activation pH and infectivity in ferrets. The H1N1pdm with K47 in HA2 is consistently both more acid stable *in vitro* and more infectious than viruses with E47 in ferrets, indicating that a highly acid stable virus is more fit in the acidic environment of the human respiratory tract. The FID_50_ value of H1N1pdm obtained herein is consistent with our previous study, in which we also showed that the H1N1pdm virus is more infectious than the previously circulating seasonal H1N1 virus [36]. A recent 2012 H1N1pdm strain was reported to have a lower fusion pH compared to Cal/09 [10]. These studies indicate that a lower fusion pH is often associated with adaptation to humans. The various challenges and lessons learned from the 2009 H1N1pdm will enable us to better understand cross-species virus adaptation from animal hosts to humans and rapidly respond to future pandemics.

## Materials and Methods

### Ethics statement

The ferret studies were conducted in an AAALAC accredited facility under a specified protocol (ACF-12-004) as approved by MedImmune's Institutional Animal Care and Use Committee (IACUC). MedImmune is registered with the United States Department of Agriculture (USDA) and applies the standards for the institutional animal care and use program as outlined in the Guide for the Care and Use of Laboratory Animals (Guide), Eighth Edition, National Research Council (NRC), 2011.

### Cells and viruses

Madin-Darby Canine Kidney (MDCK) cells, Vero cells and 293T cells were grown in Dulbecco modified Eagle medium (DMEM) containing 10% fetal bovine serum (FBS). The recombinant influenza viruses used in this study were generated by plasmid rescue as previously described and propagated in 10- to 11-day-old embryonated chicken eggs [37]. The QuikChange® Site-directed Mutagenesis kit (Agilent Technologies, Santa Clara, CA) was used to introduce changes in the HA plasmids. Recombinant wild type (wt) A/California/7/2009 (Cal/09) and wt A/Brisbane/10/2010 (Bris/10) reassortants containing the Bris/10 HA and NA and the 6 internal protein gene segments of Cal/09 were made by reverse genetics. The 6∶2 reassortant vaccine viruses contain the corresponding wt HA and NA protein genes and the 6 internal protein genes from the A/AnnArbor/6/60 (AA *ca*, H2N2) [5], [37].

### Fusion assay

HA protein-mediated membrane fusion was assessed by transient expression in 293T cells. The Green Florescent Protein (GFP) and HA genes were cloned into each cloning site of a dual expression vector pVitro2-neo-mcs (Invivogen, San Diego, CA). Point mutations were introduced into the HA by site-directed mutagenesis and confirmed by sequencing. The 293T cells were transfected with 1 µg of the HA/GFP plasmid using Lipofectamine 2000 (Invitrogen, Grand Island, NY). Twenty-four hours post transfection, cells were carefully washed one time with PBS, followed by 10 min of treatment with a trypsin-like enzyme, TrypLE (1∶5) (Gibco, Grand Island, NY), at 37°C to cleave the HA0 protein. The TrypLE was then inactivated by the addition of 10% FBS in PBS, and the transfected cells were incubated with different pH buffers ranging from 5.0 to 7.2 for 5 minutes and neutralized by the addition of DMEM/10% FBS. After incubating for 2 hours, the fused cells were monitored by GFP fluorescence using the Nikon Eclipse Ti microscope. The expression of the HA protein in transfected 293T cells was examined by Western Blot using influenza-specific antibodies. Band density was quantitated using the ImageQuant LAS 4000 Luminescent Image Analyzer (GE Healthcare).

The formation of syncytia after viral infection was evaluated by infecting monolayers of Vero cells with a multiplicity of infection (MOI) of 4.0 at 37°C for 14 hours. The infected cells were treated with PBS/TrypLE and exposed to buffers of different pH as described above. The cells were stained with the Hema-3-Stat kit (Fisher, Pittsburg, PA), and syncytia formation was examined by a light microscope.

### Acid stability studies

The acid stability of the viruses was measured by determining viral infectivity after acid treatment. Viruses were initially diluted 10-fold in PBS. The pH of the diluted viruses was lowered by careful, dropwise addition with 0.1M Citric Acid until the desired pH was reached. The viruses were then incubated at 37°C for 1 hour. The titers of acid treated viruses were determined by TCID_50_ assay in MDCK cells.

### Growth kinetics

Cells were infected with each virus at an MOI of 0.004 for 1 hour, washed twice with PBS and then incubated at 37°C for 4 days in minimum essential medium (MEM) containing 1∶40 TrypLE. The culture supernatants were collected daily, and the virus was titrated by 50% tissue culture infectious dose (TCID_50_) in MDCK cells.

### Thermal stability studies

The heat stability of the HA protein was measured by determining the loss of the hemagglutination (HA) titer after incubating the viruses at temperatures between 50°C and 65°C for 0 to 240 min. The HA titer was measured pre- and post-incubation. The evaluation of viral stability at room temperature (26°C for 35 days) and 4°C (14 weeks) was conducted with the sucrose purified virus in the vaccine virus formulation buffer, 1× SP-cGAG (Sucrose-Phosphate+1% concentrated Gelatin-Arginine-Glutamate). Duplicate aliquots were removed at various time-points and stored at −80°C prior to viral titration by TCID_50_ assay in MDCK cells.

### Ferret studies

The infectivity of the H1N1pdm wt virus was determined using 9- to 12-week-old male and female ferrets from Simonsen Laboratories (Gilroy, CA). The ferrets were individually housed and inoculated with PBS or 10, 100, or 1000 plaque forming units (PFU) of the indicated viruses intranasally. The body weight and temperature of the ferrets were monitored and nasal washes were collected daily. On day 3 after infection, the ferrets were euthanized and the nasal turbinates (NT) and lungs were harvested. The virus titer in the nasal washes, lungs and NT was determined by the TCID_50_ assay in MDCK cells and expressed as log_10_TCID_50_/gram of tissue. The ferret 50 percent infectious dose (FID_50_) was calculated by the Spearman-Karber method.

## References

[1] Garten RJ, Davis CT, Russell CA, Shu B, Lindstrom S, et al (2009) Antigenic and genetic characteristics of swine-origin 2009 A(H1N1) influenza viruses circulating in humans. Science 325: 197–201. 19465683 10.1126/science.1176225 PMC3250984

[2] WHO (2010) WHO global alert and response: pandemic (H1N1) 2009- update 112, weekly update.

[3] Dawood FS, Iuliano AD, Reed C, Meltzer MI, Shay DK, et al (2012) Estimated global mortality associated with the first 12 months of 2009 pandemic influenza A H1N1 virus circulation: a modelling study. Lancet Infect Dis 12: 687–695. 22738893 10.1016/S1473-3099(12)70121-4

[4] Hancock K, Veguilla V, Lu X, Zhong W, Butler EN, et al (2009) Cross-reactive antibody responses to the 2009 pandemic H1N1 influenza virus. N Engl J Med 361: 1945–1952. 19745214 10.1056/NEJMoa0906453

[5] Chen Z, Wang W, Zhou H, Suguitan ALJ, Shambaugh C, et al (2010) Generation of live attenuated novel influenza virus A/California/7/09 (H1N1) vaccines with high yield in embryonated chicken eggs. J Virol 84: 44–51. 19864389 10.1128/JVI.02106-09 PMC2798434

[6] Wang W, Suguitan AL Jr, Zengel J, Chen Z, Jin H (2012) Generation of recombinant pandemic H1N1 influenza virus with the HA cleavable by bromelain and identification of the residues influencing HA bromelain cleavage. Vaccine 30: 872–878. 22172510 10.1016/j.vaccine.2011.11.101

[7] Imai M, Kawaoka Y (2012) The role of receptor binding specificity in interspecies transmission of influenza viruses. Curr Opin Virol 2: 160–167. 22445963 10.1016/j.coviro.2012.03.003 PMC5605752

[8] Bullough PA, Hughson FM, Skehel JJ, Wiley DC (1994) Structure of influenza haemagglutinin at the pH of membrane fusion. Nature 371: 37–43. 8072525 10.1038/371037a0

[9] DuBois RM, Zaraket H, Reddivari M, Heath RJ, White SW, et al (2011) Acid stability of the hemagglutinin protein regulates H5N1 influenza virus pathogenicity. PLoS Pathog 7: e1002398. 22144894 10.1371/journal.ppat.1002398 PMC3228800

[10] Galloway SE, Reed ML, Russell CJ, Steinhauer DA (2013) Influenza HA subtypes demonstrate divergent phenotypes for cleavage activation and pH of fusion: implications for host range and adaptation. PLoS Pathog 9: e1003151. 23459660 10.1371/journal.ppat.1003151 PMC3573126

[11] Imai M, Watanabe T, Hatta M, Das SC, Ozawa M, et al (2012) Experimental adaptation of an influenza H5 HA confers respiratory droplet transmission to a reassortant H5 HA/H1N1 virus in ferrets. Nature 486: 420–428. 22722205 10.1038/nature10831 PMC3388103

[12] Koerner I, Matrosovich MN, Haller O, Staeheli P, Kochs G (2012) Altered receptor specificity and fusion activity of the haemagglutinin contribute to high virulence of a mouse-adapted influenza A virus. J Gen Virol 93: 970–979. 22258863 10.1099/vir.0.035782-0

[13] Zaraket H, Bridges OA, Russell CJ (2013) The pH of activation of the hemagglutinin protein regulates H5N1 influenza virus replication and pathogenesis in mice. J Virol 87: 4826–4834. 23449784 10.1128/JVI.03110-12 PMC3624295

[14] Barr IG, Cui L, Komadina N, Lee RT, Lin RT, et al (2010) A new pandemic influenza A(H1N1) genetic variant predominated in the winter 2010 influenza season in Australia, New Zealand and Singapore. Euro Surveill 15: pii: 19692. 10.2807/ese.15.42.19692-en 21034722

[15] Ikonen N, Haanpaa M, Ronkko E, Lyytikainen O, Kuusi M, et al (2009) Genetic diversity of the 2009 pandemic influenza A(H1N1) viruses in Finland. PLoS One 5: e13329. 10.1371/journal.pone.0013329 PMC2958116 20975994

[16] Mak GC, Leung CK, Cheng KC, Wong KY, Lim W (2011) Evolution of the haemagglutinin gene of the influenza A(H1N1)2009 virus isolated in Hong Kong, 2009–2011. Euro Surveill 16: pii: 19807. 21392488

[17] Maurer-Stroh S, Lee RT, Eisenhaber F, Cui L, Phuah SP, et al (2010) A new common mutation in the hemagglutinin of the 2009 (H1N1) influenza A virus. PLoS Curr 2: RRN1162. 20535229 10.1371/currents.RRN1162 PMC2880458

[18] Xu R, Ekiert DC, Krause JC, Hai R, Crowe JEJ, et al (2010) Structural basis of preexisting immunity to the 2009 H1N1 pandemic influenza virus. Science 328: 357–360. 20339031 10.1126/science.1186430 PMC2897825

[19] Yang H, Carney P, Stevens J (2010) Structure and Receptor binding properties of a pandemic H1N1 virus hemagglutinin. PLoS Curr Mar 22: RRN1152. 10.1371/currents.RRN1152 PMC2846141 20352039

[20] Murakami S, Horimoto T, Ito M, Takano R, Katsura H, et al (2011) Enhanced growth of influenza vaccine seed viruses in vero cells mediated by broadening the optimal pH range for virus membrane fusion. J Virol 86: 1405–1410. 22090129 10.1128/JVI.06009-11 PMC3264368

[21] Carr CM, Chaudhry C, Kim PS (1997) Influenza hemagglutinin is spring-loaded by a metastable native conformation. Proc Natl Acad Sci U S A 94: 14306–14313. 9405608 10.1073/pnas.94.26.14306 PMC24954

[22] Haywood AM, Boyer BP (1986) Time and temperature dependence of influenza virus membrane fusion at neutral pH. J Gen Virol 67 (Pt 12) 2813–2817. 3794667 10.1099/0022-1317-67-12-2813

[23] Ruigrok RW, Martin SR, Wharton SA, Skehel JJ, Bayley PM, et al (1986) Conformational changes in the hemagglutinin of influenza virus which accompany heat-induced fusion of virus with liposomes. Virology 155: 484–497. 3788061 10.1016/0042-6822(86)90210-2

[24] Strengell M, Ikonen N, Ziegler T, Julkunen I (2011) Minor changes in the hemagglutinin of influenza A(H1N1)2009 virus alter its antigenic properties. PLoS One 6: e25848. 22022458 10.1371/journal.pone.0025848 PMC3191144

[25] Steinhauer DA, Martín J, Lin YP, Wharton SA, Oldstone MB, et al (1996) Studies using double mutants of the conformational transitions in influenza hemagglutinin required for its membrane fusion activity. Proc Natl Acad Sci U S A 93: 12873–12878. 8917512 10.1073/pnas.93.23.12873 PMC24013

[26] Daniels RS, Downie JC, Hay AJ, Knossow M, Skehel JJ, et al (1985) Fusion mutants of the influenza virus hemagglutinin glycoprotein. Cell 40: 431–439. 3967299 10.1016/0092-8674(85)90157-6

[27] Giannecchini S, Campitelli L, Calzoletti L, De Marco MA, Azzi A, et al (2006) Comparison of in vitro replication features of H7N3 influenza viruses from wild ducks and turkeys: potential implications for interspecies transmission. J Gen Virol 87: 171–175. 16361429 10.1099/vir.0.81187-0

[28] Lin YP, Wharton SA, Martín J, Skehel JJ, Wiley DC, et al (1997) Adaptation of egg-grown and transfectant influenza viruses for growth in mammalian cells: selection of hemagglutinin mutants with elevated pH of membrane fusion. Virology 233: 402–410. 9217063 10.1006/viro.1997.8626

[29] Reed ML, Bridges OA, Seiler P, Kim JK, Yen HL, et al (2010) The pH of activation of the hemagglutinin protein regulates H5N1 influenza virus pathogenicity and transmissibility in ducks. J Virol 84: 1527–1535. 19923184 10.1128/JVI.02069-09 PMC2812356

[30] Reed ML, Yen HL, DuBois RM, Bridges OA, Salomon R, et al (2009) Amino acid residues in the fusion peptide pocket regulate the pH of activation of the H5N1 influenza virus hemagglutinin protein. J Virol 83: 3568–3580. 19193808 10.1128/JVI.02238-08 PMC2663236

[31] Steinhauer DA, Wharton SA, Skehel JJ, Wiley DC, Hay AJ (1991) Amantadine selection of a mutant influenza virus containing an acid-stable hemagglutinin glycoprotein: evidence for virus-specific regulation of the pH of glycoprotein transport vesicles. Proc Natl Acad Sci U S A 88: 11525–11529. 1763066 10.1073/pnas.88.24.11525 PMC53168

[32] Krenn BM, Egorov A, Romanovskaya-Romanko E, Wolschek M, Nakowitsch S, et al (2011) Single HA2 mutation increases the infectivity and immunogenicity of a live attenuated H5N1 intranasal influenza vaccine candidate lacking NS1. PLoS One 6: e18577. 21490925 10.1371/journal.pone.0018577 PMC3072404

[33] Fukuyama S, Kawaoka Y (2011) The pathogenesis of influenza virus infections: the contributions of virus and host factors. Curr Opin Immunol 23: 481–486. 21840185 10.1016/j.coi.2011.07.016 PMC3163725

[34] Smeenk CA, Brown EG (1994) The influenza virus variant A/FM/1/47-MA possesses single amino acid replacements in the hemagglutinin, controlling virulence, and in the matrix protein, controlling virulence as well as growth. J Virol 68: 530–534. 8254767 10.1128/jvi.68.1.530-534.1994 PMC236317

[35] Smeenk CA, Wright KE, Burns BF, Thaker AJ, Brown EG (1996) Mutations in the hemagglutinin and matrix genes of a virulent influenza virus variant, A/FM/1/47-MA, control different stages in pathogenesis. Virus Res 44: 79–95. 8879138 10.1016/0168-1702(96)01329-9

[36] Suguitan ALJ, Zengel JR, Jacobson S, Gee S, Cetz J, et al (2013) Influenza H1N1pdm-specific maternal antibodies offer limited protection against wild-type virus replication and influenced influenza vaccination in ferrets. Influenza Other Respi Viruses in press. 10.1111/irv.12220 PMC4186464 24734293

[37] Jin H, Lu B, Zhou H, Ma C, Zhao J, et al (2003) Multiple amino acid residues confer temperature sensitivity to human influenza virus vaccine strains (FluMist) derived from cold-adapted A/Ann Arbor/6/60. Virology 306: 18–24. 12620793 10.1016/s0042-6822(02)00035-1

